# Case report: Surgical repair of congenitally corrected transposition of the great arteries with the guidance of three-dimensional printing

**DOI:** 10.3389/fcvm.2022.1101929

**Published:** 2023-01-06

**Authors:** Yanchun Zhang, Yongnan Li, YuMei Ma, Yalin Wei, Fengxiao He, Yilin Zhu, Weixin Lu, Yinglu Zhao, Xiangyang Wu

**Affiliations:** Department of Cardiovascular Surgery, The Second Affiliated Hospital of Lanzhou University, Lanzhou, China

**Keywords:** congenitally corrected transposition of the great arteries (ccTGA), membranous ventricular septal aneurysm, atrial septal defect (ASD), pulmonary stenosis (PS), three-dimensional printing

## Abstract

A 10-year-old girl presented with obvious cyanosis, and the saturation of arterial blood oxygen (SpO_2_) was decreased to 60.5% in the outpatient examination. Computed tomography angiography (CTA) and echocardiography suggested congenitally corrected transposition of the great arteries (ccTGAs), membranous ventricular septal aneurysm (MVSA), atrial septal defect (ASD), severe pulmonary stenosis (PS), and severe tricuspid regurgitation (TR). Due to the complex pathological anatomical structures, the three-dimensional printed model was used for preoperative assessment. After a comprehensive evaluation was completed, the operation was performed by physiological correction under cardiopulmonary bypass, including the resection of MVSA, repair using the bovine pericardial patch for ASD, and linear valvuloplasty of the tricuspid valve. Due to the special anatomical structures of ccTGA, PS was treated by extracardiac pipe technique. After the operation, the patient recovered well, cyanosis disappeared, SpO_2_ was up to 96%, and the extracardiac pipe was well-functioning without regurgitation or obstruction.

## Introduction

Congenitally corrected transposition of the great arteries (ccTGAs) is a complex pathological malformation, accounting for 0.5–1% of congenital heart diseases (CHDs), among which 80–90% of children are complicated with ventricular septal defects, pulmonary stenosis (PS), complete conduct block, and other malformations (1). During embryonic development, the deformed bending of the blood vessels may lead to abnormal connections between the atrium ventricle and ventricle artery. The treatment and management of ccTGA remain a challenge. Surgical strategies include physiological correction and anatomical correction. Physiological correction fixes the defect in maintaining the right ventricle as a systemic ventricle, increasing the long-term risk of systolic dysfunction, especially when associated with tricuspid regurgitation (TR). In addition, anatomical correction restores atrioventricular and ventriculoarterial coherence in systemic circulation by combining different surgical procedures (2). The optimal surgical strategy for ccTGA treatment depends on the specific anatomical structures and requires sufficient individualized consideration. Nowadays, cardiovascular three-dimensional (3D) printing has played a critical role in preoperative evaluation and surgical strategy formulation (3, 4). In this case report, we introduce the treatment of ccTGA under the guidance of 3D printing technology.

## Baseline characteristics and preoperative imaging evaluation

**FIGURE 1:**
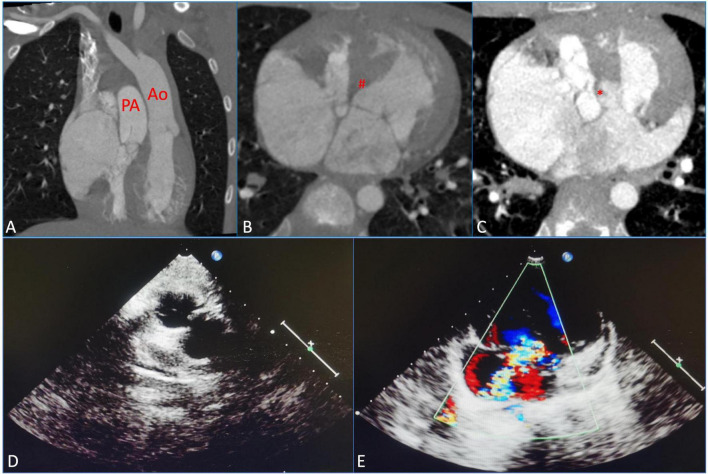
Preoperative measurements using computed tomography, angiography, and echocardiography were assessed before the surgical repair. **(A)** Congenitally corrected transposition of the great arteries (ccTGAs). **(B)** Membranous ventricular septal aneurysm (MVSA). (#) Represents MVSA. **(C)** Atrial septal defect (ASD). (*) Represents ASD. **(D)** Severe pulmonary stenosis (PS). **(E)** Severe tricuspid regurgitation (TR). PA, pulmonary artery; Ao, aorta.

A 10-year-old girl had heart murmurs for 8 years with obvious cyanosis. The oxygen pressure of arterial blood (PO_2_) was 33.4 mmHg, and the saturation of arterial blood oxygen (SpO_2_) was decreased to 60.5%. Preoperative computed tomography angiography (CTA) suggested ccTGA, severe PS, and membranous ventricular septal aneurysm (MVSA) (maximum diameter was up to 14 mm) with multiple defects, atrial septal defect (ASD) (the length of ASD was 1.5 cm) (Figures 1A–C). Preoperative echocardiography (ECG) displayed severe PS (the inner diameter of the pulmonary artery was 22 mm) and severe TR (the regurgitation volume was 8.0 mL) (Figures 1D, E). The left ventricular systolic function was normal [left ventricular end-diastolic volume index (LVEDVI) was 47 mL/m^2^].

## Three-dimensional printed model and preoperative evaluation

**FIGURE 2:**
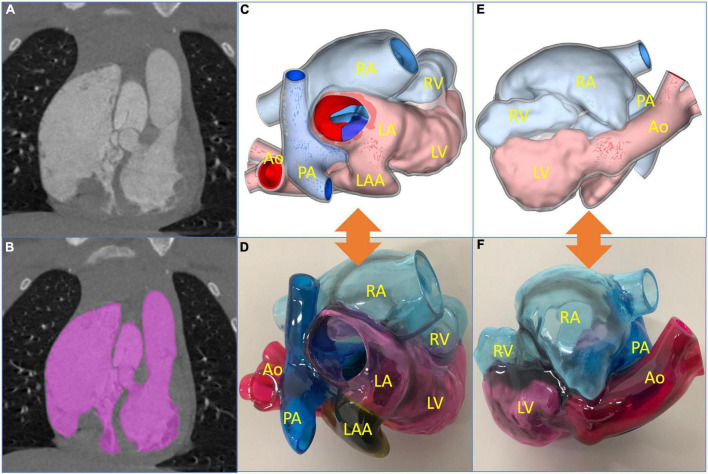
Reconstructed process of three-dimensional (3D) printed model and preoperative evaluation. **(A)** Original computed tomography angiography data. **(B)** Edition of the mask. **(C,D)** The 3D reconstructed and printed models are shown in the front view, respectively. **(E,F)** The 3D reconstructed and printed models are shown in the rearview, respectively. In the 3D printed model, the red part is the aorta (Ao), the blue part is the pulmonary artery (PA), the yellow part is the left atrial appendage (LAA), the pink part is the left atrium (LA) and left ventricle (LV), and the light blue part is right atrium (RA) and right ventricle (RV).

Digital imaging and communication of medicine (DICOM) format of the patient CTA data was imported into Materialize Mimics version 21.0 (Leuven, Belgium), and the 3D reconstructed model was segmented using the threshold segmentation function (Figure 2A). Then, the acquired 3D reconstructed model of the heart was digitally removed, trimmed, smoothened, and repaired in Materialize 3-matic (Leuven, Belgium) to restore the complete anatomical structures of the heart (Figure 2B). Standard tessellation language (STL) file of 3D reconstruction was exported to Stratasys Polyjet 850 multi-material full-color 3D printer (Figures 2C, E). The digital acrylonitrile butadiene styrene was used to print the model, different anatomical structures were matched with different colors, and then the 3D printed whole-heart model was obtained (Figures 2D, F).

According to the 3D printed model, surgeons may intuitively understand the specific anatomical structures and adjacent relationships of the heart. In addition, the diameter and length of the extracardiac pipe required during the operation may be measured on the model, so as to plan the operation more accurately and effectively. After a comprehensive evaluation, the team decided to perform surgical repair of physiological correction.

## Surgical steps

**FIGURE 3:**
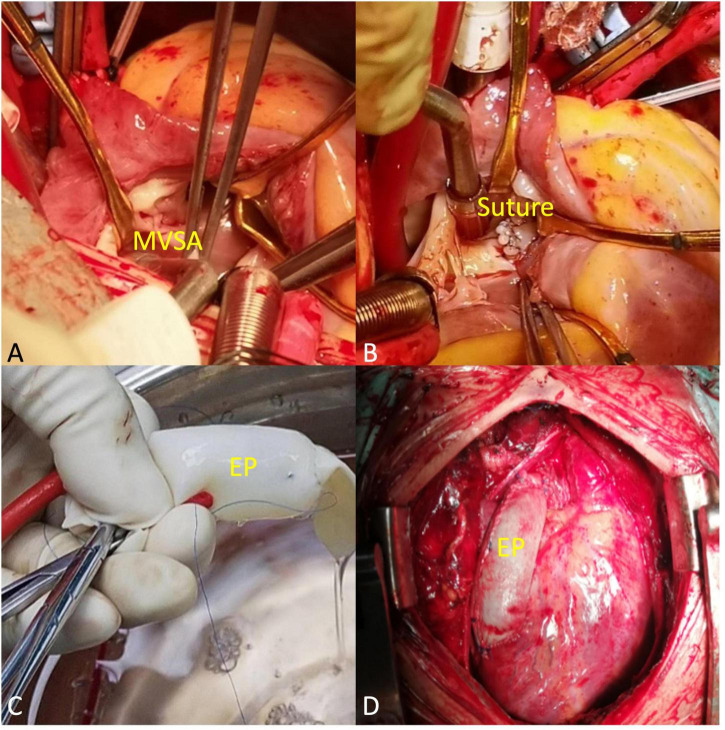
Main procedural steps. **(A)** The membranous ventricular septal aneurysm (MVSA) was positioned toward the right ventricular outflow tract (RVOT). The arrowheads to MVSA. **(B)** MVSA was resected, and the residual membranous part of the interventricular septum was directly stitched. The arrowheads to the suture. **(C)** The extracardiac pipe (EP) made by a bovine pericardial patch was tested before connection. **(D)** EP completed the connection between the right ventricle and the pulmonary artery.

**FIGURE 4:**
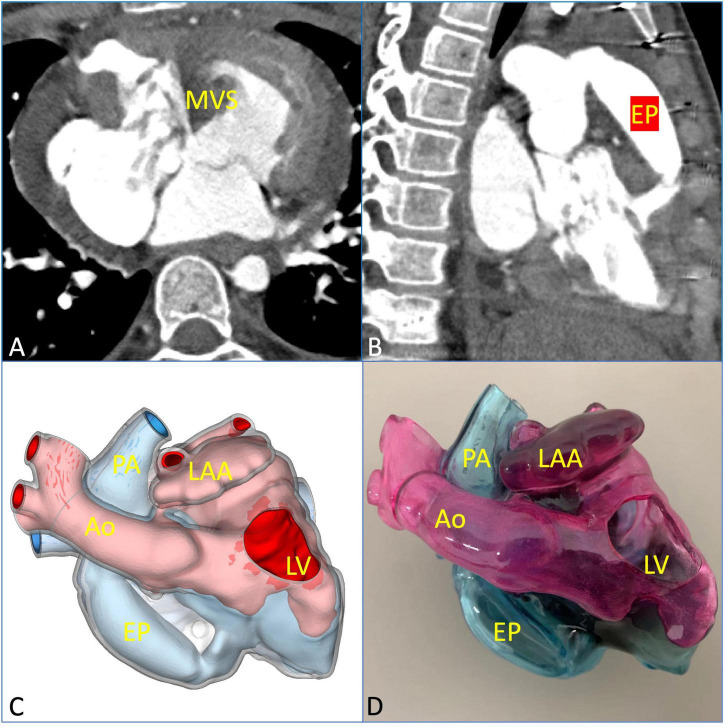
Postoperative evaluation using multimodal imaging verified the curative effect of the surgical repair. **(A)** The residual membranous part of the interventricular septum was in a good position and well-functioning. **(B)** The extracardiac pipe functioned well. **(C,D)** The postoperative 3D printed model was observed clearly to verify the effect of the operation. EP, extracardiac pipe.

A midsternal incision was made, and aorta-superior and inferior vena cava cardiopulmonary bypass was established. The small right ventricle was revealed with obvious stenosis of the right ventricular outflow tract (RVOT) and PV. MVSA was formed, and the aneurysm presented like protrusion to RVOT (Figure 3A). The stenosis was aggravated during systole without ventricular septal defect, and the tricuspid valve (TV) was parallel to RVOT and well developed. MVSA was removed, and the residual membranous part of the interventricular septum was directly stitched (Figure 3B). The anterior leaflet of the TV was sutured with a spacer, and ASD was repaired with a patch. Due to the special adjacent relationships of PV, the connection between the right ventricle and pulmonary artery was established by using the extracardiac pipe technique. The pipe was made by a bovine pericardial patch with a diameter close to that of the aorta. The method was as follows: Three leaflets of the same size were cut and sewn parallel to the bovine pericardial patch. Then, the bovine pericardial patch was rolled into the tube shape and sutured to form a self-made pipe, which was used in RVOT reconstruction (Figures 3C, D). After the pipe reconnected, the heart returned to the sinus rhythm. The patient recovered well after the operation, and cyanosis disappeared before discharge. There was no residual regurgitation in the atrial septum and only mild TR. The residual membranous part of the interventricular septum was positioned well (Figure 4A). The extracardiac pipe was well-functioning without regurgitation or stenosis (Figure 4B). The postoperative 3D reconstructed and printed model showed the morphology of the anatomical structures, indicating that the operation achieved good results (Figures 4C, D).

## Discussion

Due to the abnormal connections of the heart cavities, ccTGA often leads to the reversal of the left and right ventricles’ load, which results in left/right ventricular dysfunction and heart failure. Complicated intracardiac malformations have always been a challenge for surgeons. Traditional corrective surgery in the treatment of ccTGA has an ordinary long-term prognosis and has been gradually abandoned. Double switch surgery and Fontan surgery are the most commonly used surgical methods at present (5). Left ventricular function degeneration is common in ccTGA. Studies have reported that double-switch surgery was often followed by pulmonary artery constriction surgery (6). However, long-term outcomes of pulmonary artery constriction treatment showed higher mortality and worse cardiac function, which may be related to a left ventricular failure caused by excessive constriction (7). Our case was complicated with severe PS, ASD, and a smaller left ventricle, which was similar to the mechanism of excessive pulmonary constriction and did not meet the indications for double switch surgery. Therefore, the purpose of the operation was to improve the function and transition to heart transplantation or other types of surgery. External pipe connection between the right ventricle and pulmonary artery is one of the surgical methods in complex CHD surgeries (8). There are few reports on the self-made extracardiac pipe of the bovine pericardial patch used in this operation. The advantage is that different sizes of pipes can be made according to the actual needs during operations, which is relatively simple and has good short- and midterm outcomes. However, long-term studies are needed to carry out to show further results.

With the continuous development of cardiovascular 3D printing technology, surgeons may use 3D printed models for preoperative evaluation, which plays an important role in assisting and guiding the accurate formulation of surgical plans and improving the success rate of operations (9). Preoperative familiarity with cardiac morphology significantly facilitates the operation. Before the operation, the CTA data of the patient were evaluated in detail, the important anatomical structures and adjacent relationships were reconstructed, and 3D models were printed. In addition, the 3D printed model was used to intuitively understand the patient’s specific anatomical structures and reasonably plan the surgical strategy. With the guidance of 3D printing, the operation was successful, the patient recovered quite well, and cyanosis disappeared before discharge. There was no residual regurgitation in the atrial septum and only mild TR. The extracardiac pipe was well-functioning without regurgitation or stenosis. Furthermore, it is believed that with the progress of minimally invasive interventional technology for structural heart disease, more optimized surgical designs are bound to emerge, which will bring more satisfactory treatment plans for patients.

In conclusion, the use of a bovine pericardial patch for the external pipe connection between the right ventricle and pulmonary artery may achieve good results. Meanwhile, on the basis of accurate preoperative evaluation with various medical imaging methods, the 3D printed model is used to complete the formulation of the surgery plan. With the continuous development of material science and imaging technology, cardiovascular 3D printing technology is gradually mature and perfect, which not only intuitively shows the anatomical structures with lesions but also simulates the bench test.

## Data availability statement

The original contributions presented in this study are included in this article/supplementary materials, further inquiries can be directed to the corresponding author.

## Ethics statement

The studies involving human participants were reviewed and approved by the Ethical Committee of The Second Affiliated Hospital of Lanzhou University (D2021-283). Written informed consent to participate in this study was provided by the participants’ legal guardian/next of kin. Written informed consent was obtained from the individual(s), and minor(s)’ legal guardian/next of kin, for the publication of any potentially identifiable images or data included in this article.

## Author contributions

YaZ and YL: concept and design. YM, YW, FH, YilZ, WL, and YinZ: acquisition, analysis, and interpretation of data. YaZ: drafting of the manuscript. XW: supervision. All authors had full access to all of the data in the study, took responsibility for the integrity of the data and the accuracy of the data analysis, and critical revised the manuscript for important intellectual content.
